# Hyperdense artery sign, symptomatic infarct swelling and effect of alteplase in acute ischaemic stroke

**DOI:** 10.1136/svn-2020-000569

**Published:** 2020-11-27

**Authors:** Simiao Wu, Grant Mair, Geoff Cohen, Zoe Morris, Anders von Heijne, Nick Bradey, Lesley Cala, Andre Peeters, Andrew J Farrall, Alessandro Adami, Gillian Potter, Ming Liu, Richard I Lindley, Peter A G Sandercock, Joanna M Wardlaw, IST-3 Collaborative Group

**Affiliations:** 1 Department of Neurology, West China Hospital, Sichuan University, Chengdu, Sichuan, China; 2 Centre for Clinical Brain Sciences, The University of Edinburgh, Edinburgh, UK; 3 Department of Clinical Sciences, Danderyd Hospital, Stockholm, Sweden; 4 Neuroradiology, James Cook University Hospital, South Tees Hospital NHS Trust, Middlesbrough, UK; 5 Division of Pathology and Laboratory Medicine, School of Medicine, The University of Western Australia, Perth, Western Australia, Australia; 6 Department of Neurology, Cliniques universitaires Saint-Luc, Bruxelles, Belgium; 7 Stroke Center, IRCCS Sacro Cuore Don Calabria, Verona, Italy; 8 Department of Neuroradiology, Manchester Centre for Clinical Neurosciences, Salford Royal NHS Foundation Trust, Salford, Manchester, UK; 9 Westmead Applied Research Centre, Westmead Clinical School, The University of Sydney, Sydney, New South Wales, Australia; 10 The George Institute for Global Health, Newtown, New South Wales, Australia; 11 UK Dementia Research Institute Centre at the University of Edinburgh, Edinburgh, UK

**Keywords:** stroke, thrombolysis, complication

## Abstract

**Background:**

Alteplase improves functional outcomes of patients with acute ischaemic stroke, but its effects on symptomatic infarct swelling, an adverse complication of stroke and the influence of CT hyperdense artery sign (HAS) are unclear. This substudy of the Third International Stroke Trial aimed to investigate the association between HAS and symptomatic infarct swelling and effect of intravenous alteplase on this association.

**Methods:**

We included stroke patients whose prerandomisation scan was non-contrast CT. Raters, masked to clinical information, assessed baseline (prerandomisation) and follow-up (24–48 hours postrandomisation) CT scans for HAS, defined as an intracranial artery appearing denser than contralateral arteries. Symptomatic infarct swelling was defined as clinically significant neurological deterioration ≤7 days after stroke with radiological evidence of midline shift, effacement of basal cisterns or uncal herniation.

**Results:**

Among 2961 patients, HAS presence at baseline was associated with higher risk of symptomatic infarct swelling (OR 2.21; 95% CI 1.42 to 3.44). Alteplase increased the risk of swelling (OR 1.69; 95% CI 1.11 to 2.57), with no difference between patients with and those without baseline HAS (p=0.49). In patients with baseline HAS, alteplase reduced the proportion with HAS at follow-up (OR 0.67; 95% CI 0.50 to 0.91), where HAS disappearance was associated with reduced risk of swelling (OR 0.25, 95% CI 0.14 to 0.47).

**Conclusion:**

Although alteplase was associated with increased risk of symptomatic infarct swelling in patients with or without baseline HAS, it was also associated with accelerated clearance of HAS, which in return reduced swelling, providing further mechanistic insights to underpin the benefits of alteplase.

## Introduction

Symptomatic infarct swelling is a devastating neurological complication of acute ischaemic stroke.^1^ Currently, there is insufficient evidence to inform its prevention and management in practice,^2^ although observational studies have shown that persistent arterial occlusion was associated with more infarct swelling and reperfusion appeared to reduce the risk of swelling.^3^ The hyperdense artery sign (HAS) on non-contrast CT is a widely accessible imaging sign in stroke practice,^4 5^ which is highly specific and moderately sensitive for detection of intracranial arterial obstruction by thrombus.^6^ However, the relationship between HAS and swelling is less clear as divergent findings were reported in previous studies.^7–12^

Although there are concerns that reperfusion therapies might increase infarct swelling, the Multicentre Randomised Clinical Trial of Endovascular Treatment for Acute Ischaemic Stroke in the Netherlands (MR CLEAN) showed that successful reperfusion by mechanical clot retrieval reduced the development of midline shift.^13^ Nevertheless, the impact of arterial recanalisation by intravenous thrombolysis on the development of severe infarct swelling is unknown.^14^ In the Third International Stroke Trial (IST-3), intravenous alteplase increased HAS clearance, and the disappearance of HAS was associated with better functional outcome.^15^ Therefore, intravenous thrombolysis is a promising therapy for patients with HAS, particularly where thrombectomy is not available.

In this study, we used data from IST-3 to investigate whether the presence of HAS on baseline CT predicted development of symptomatic infarct swelling and the effect of intravenous alteplase on this association.

## Methods

### Third International Stroke Trial

IST-3 was an international, multicentre, prospective, randomised, open- blinded endpoint trial in acute ischaemic stroke comparing intravenous alteplase versus control, recruiting between May 2000 and July 2011. IST-3 is registered, ISRCTN25765518. The detailed study design and methods of the IST-3 have been described elsewhere.^16–19^ Briefly, patients were eligible if they had clinically definite acute stroke of any severity (assessed prior to randomisation with the National Institute of Health Stroke Scale (NIHSS)), age ≥18 years, for whom CT or MRI had excluded intracranial haemorrhage and any structural stroke mimics, and the treatment could be started within 6 hours of stroke onset, without clear indications for, or contraindications to, intravenous thrombolysis with alteplase. In total, 3035 patients were randomised to receive standard care plus intravenous alteplase (0.9 mg/kg) or standard care alone. No intra-arterial therapy was used. Primary outcome was the functional status at 6 months assessed with the Oxford Handicap Scale. Results were analysed on an intention-to-treat basis. Collaborators and funders of IST-3 are listed in the online supplemental material 1.

10.1136/svn-2020-000569.supp1 Supplementary data

### Imaging protocol and analysis

The IST-3 imaging protocol has been described previously.^16 20^ All patients had a brain CT or MRI scan before randomisation and a follow-up scan at 24–48 hours after randomisation. A repeat brain scan was required if the patient deteriorated neurologically or intracranial haemorrhage was suspected for any reason. Non-contrast CT scans were required to cover the brain from foramen magnum to vertex, with maximum slice thickness of 4–5 mm through the posterior fossa and 8–10 mm for the cerebral hemispheres, with no slice gap. Scans were windowed on 80 Hounsfield Units (HU) width and centre level of 35–40 HU. A centralised panel of neurologists and neuroradiologists experienced in acute stroke imaging evaluated all relevant CT scans using a validated, prespecified rating proforma (www.ed.ac.uk/clinical-sciences/edinburgh-imaging/research/analysis-and-processing/image-analysis-tools-downloads/all-the-edinburgh-imaging-rating-tools).^5 16 21^ All raters were blinded to clinical data and imaging at other time points.

HAS presence was determined visually on non-contrast CT on both baseline (prerandomisation) and follow-up (24–48 hour postrandomisation) scans. No objective HU measurements were made, as in prior research, we found that this approach did not materially increase sensitivity or specificity for acute thrombus.^22 23^ The raters identified HAS by deciding if an intracranial artery appeared denser than adjacent arteries or than equivalent contralateral arteries, but not due to calcification, at the following locations: internal carotid artery, mainstem or sylvian branches of middle cerebral artery (MCA), anterior cerebral artery (ACA), posterior cerebral artery (PCA), vertebral or basilar arteries. In a large observer reliability study based on the IST-3 imaging data, the inter-rater and intra-rater reliability of HAS identification by Krippendorff α was 0.59 and 0.58, respectively.^6^ The status of HAS was recorded as ‘persistent’ for HAS present on both baseline and follow-up CT; ‘disappeared’ for HAS present at baseline but not follow-up; ‘newly developed’ for HAS not present on baseline CT but on follow-up CT’ and ‘none’ for HAS absent at both baseline and follow-up.

We assessed the extent of the acute ischaemic lesion using the IST-3 score, which includes all arterial territories and differentiates infarct extent from brain swelling.^16^ The score has been widely validated and shown to have better inter-rater reliability and dynamic range than other infarct extent scores.^16 21 24^ We condensed the full-lesion extent scores into four groups for analysis, as previously^16^: small infarcts, defined as lacunar, small cortical, small cerebellar, less than half of brainstem or less than half of the ACA or PCA territory; medium infarcts, defined as striatocapsular, the anterior or posterior half of the peripheral MCA territory or more than half of the ACA or PCA territory; large infarcts, defined as the whole of the peripheral MCA territory or all the MCA territory and very large infarcts, defined as the whole MCA and PCA territory, the whole MCA and ACA territory or all three territories.

### Definition of symptomatic infarct swelling

The outcome in this analysis was prespecified in the IST-3 study protocol,^17 19^ where symptomatic infarct swelling was defined as clinically significant neurological deterioration (as assessed and reported by trial investigators according to their local protocol) or death ≤7 days of randomisation, accompanied by evidence of significant infarct swelling as determined by the independent masked expert assessment of the scan. Significant infarct swelling was defined as any degree of the shift of the midline cerebral structures away from their usual central location in the brain, effacement of the basal cisterns or uncal herniation on a follow-up scan or finding severe infarct swelling at autopsy if not rescanned before death. The presence of some degree of haemorrhagic transformation was permitted, provided it was not considered by the expert imaging rater to be a major contributor to the mass effect.

### Statistical analysis

Patients whose prerandomisation radiological assessment was a non-contrast CT scan that had undergone blinded central review were included in this analysis. We excluded the few patients with prerandomisation MRI scans. We compared clinical characteristics between patients with and without symptomatic infarct swelling, using t-tests, Wilcoxon test or χ^2^ tests as appropriate. We performed tests of interaction to compare ORs for the association between HAS and symptomatic infarct swelling in patients with an initial NIHSS <15 versus ≥15; patients with large to very large infarcts versus small to medium infarcts and patients allocated to alteplase versus those on control. We also used logistic regression with symptomatic infarct swelling as dependent variable to calculate ORs for HAS, adjusted for the effects of patient age, NIHSS, extent of ischaemic lesion and allocation to alteplase. IST-3 was powered to detect the clinical difference in the primary outcome, and the clinical (symptomatic infarct swelling) and imaging (HAS) variables for secondary analysis were prespecified.^17 18 20^ In addition, we had been cautious with the number of variables allowed in regression analysis to satisfy the sample size criterion of ≥10 events per variable.^25 26^ Therefore, the study was anticipated to be sufficient to enable the assessment of the effects of alteplase on HAS and on infarct swelling. All analyses were performed with SAS software V.9.4 (SAS Institute, Cary, North Carolina, USA). All tests were two sided with p<0.05 as significant.

## Results

Of 3035 patients randomised in the IST-3, non-contrast CT scans were centrally reviewed for 2961 patients at baseline (18/3035 patients with baseline CT were not available for central review and MRI only was performed at baseline in the remaining 56/3035 patients) and for 2731 patients at both baseline and follow-up. HAS data were missing for one follow-up CT due to poor quality scan, thus 2730 patients had HAS data at both baseline and follow-up.

### Associations between baseline HAS and symptomatic infarct swelling

Of 2961 patients with baseline CT scans (mean age, 77.4 (SD, 12.1) years; men, 48.2%; onset to scan time, median 3 (IQR, 2–4 hours), 716 (24.2%)) demonstrated baseline HAS and 107 (3.6%) developed symptomatic infarct swelling ≤7 days after stroke onset (median, 3 (IQR 1–4) days). Patients with baseline HAS had higher NIHSS (median, 16 (IQR 10–20]) than those without HAS (median, 9 (IQR 6–16); p<0.001). Patients were more likely to develop symptomatic infarct swelling if they had baseline HAS (unadjusted OR, 4.83 (95% CI 3.25 to 7.16); p<0.001), NIHSS ≥15 (unadjusted OR, 8.82 (95% CI 5.34 to 14.56); p<0.001), large to very large infarcts (unadjusted OR, 5.76 (95% CI 3.89 to 8.52); p<0.001) and were allocated to alteplase (unadjusted OR, 1.70 (95% CI 1.14 to 2.53); p=0.008). There was no association between symptomatic infarct swelling and age, sex or time from onset to randomisation (table 1). The sensitivity and specificity of baseline HAS for predicting symptomatic infarct swelling was 0.59 (95% CI 0.50 to 0.64) and 0.77 (95% CI 0.73 to 0.82), respectively. Baseline HAS was associated with a higher risk of death ≤7 days (unadjusted OR, 2.16 (95% CI 1.66 to 2.82); p<0.001), and the development of symptomatic infarct swelling did not change the adverse association of baseline HAS with early death (p for interaction 0.11).

**Table 1:** Baseline characteristics and 7 day fatality of patients with and without symptomatic infarct swelling

	Symptomatic infarct swelling (n=107)	No symptomatic infarct swelling (n=2854)	P values
Age, years, mean (SD)	76.8 (12.5)	77.4 (12.1)	0.59
Sex, n (%)*			0.76
Male	50 (3.5)	1377 (96.5)	
Female	57 (3.7)	1477 (96.3)	
Baseline NIHSS, median (IQR)	19 (17–23)	11 (6–17)	<0.001
NIHSS, n (%)*			<0.001
NIHSS ≥15	88 (8.2)	983 (91.8)	
NIHSS <15	19 (1.0)	1871 (99.0)	
Infarct size on pre-randomisation CT, n (%)*			<0.001
Large/very large	55 (11.0)	443 (89.0)	
None/small/medium	52 (2.1)	2411 (97.9)	
Hyperdense artery sign on prerandomisation CT, n (%)*			<0.001
HAS	63 (8.8)	653 (91.2)	
No HAS	44 (2.0)	2201 (98.0)	
Allocated treatment, n (%)*			0.008
Alteplase	67 (4.5)	1417 (95.5)	
Control	40 (2.7)	1437 (97.3)	
Death in first 7 days, n (%)†	70 (65.4)	192 (6.7)	<0.001
Time from onset to randomisation, hours, median (IQR)	3.5 (2.7–4.2)	3.9 (2.9–4.9)	0.14

*The proportion of patients with/without infarct swelling in each row characteristic subgroup.

†The proportion of patients who died ≤7 days in patients with/without symptomatic infarct swelling.

HAS, hyperdense artery sign; NIHSS, National Institute of Health Stroke Scale.

In multivariable analysis, the risk of symptomatic infarct swelling increased independently with higher NIHSS, larger infarcts, presence of baseline HAS and allocation to alteplase and decreased with increasing age (table 2). The association between HAS and symptomatic infarct swelling was stronger among patients with baseline NIHSS <15 than those with NIHSS ≥15 (OR, 7.44 (95% CI 2.97 to 18.64) vs OR, 2.49 (95% CI 1.60 to 3.88); p for interaction 0.04), but this interaction between HAS and NIHSS was no longer present when a linear function of NIHSS was assessed in multivariable regression, adjusted for age, baseline HAS, large to very large infarct and allocation to alteplase (p=0.37, data not shown). The effects of alteplase (p for interaction 0.49) and of large to very large infarcts (p for interaction 0.81) on increased risk of symptomatic infarct swelling showed no significant interaction with baseline HAS presence or absence.

**Table 2:** Multivariable logistic regression for symptomatic infarct swelling

Factors	Beta	OR	95% CI	P values
Age*	−0.02	0.98	0.97 to 0.99	0.04
NIHSS*	0.14	1.15	1.11 to 1.18	<0.001
Large to very large infarct (vs small to medium infarct)	0.89	2.44	1.57 to 3.79	<0.001
Allocated alteplase (vs control)	0.53	1.69	1.11 to 2.57	0.01
HAS presence (vs absence)	0.79	2.21	1.42 to 3.44	<0.001

*Entered as linear variables, where the OR reflected the expected change in odds for one-point increase in NIHSS or 1-year increase in age.

HAS, hyperdense artery sign; NIHSS, National Institute of Health Stroke Scale.

### Effect of alteplase on the development of symptomatic infarct swelling

Of 2730 patients with information on HAS on both baseline and follow-up CT, HAS was identified on 673 (24.7%) baseline scans, of whom 60 (8.9% of 673) patients developed symptomatic infarct swelling. Alteplase increased the risk of symptomatic infarct swelling in general, and this pattern persisted regardless of changes in HAS (table 3).

**Table 3:** Effect of alteplase on the development of symptomatic infarct swelling by change in HAS between baseline and follow-up

Baseline HAS	Changes in HAS at follow-up	Alteplase n/N*	Control n/N*	Effect of alteplase on development of infarct swelling, OR (95% CI)	P value for interaction
Presence (n=673)	Persistent (n=324)	28/152	18/172	1.93 (1.02 to 3.66)	0.62; d.f.=1
	Disappeared (n=349)	9/198	5/151	1.39 (0.46 to 4.24)	
	Subtotal	37/350	23/323	1.54 (0.89 to 2.66)	
Absence (n=2057)	Newly developed (n=196)	8/90	5/106	1.97 (0.62 to 6.25)	0.98; d.f.=1
	None (n=1861)	21/957	10/904	2.01 (0.94 to 4.28)	
	Subtotal	29/1047	15/1010	1.89 (1.01 to 3.55)	
Total	NA	66/1397	38/1333	1.69 (1.13 to 2.54)	0.96; d.f.=3

*N or n=number of patients in each subgroup of morphological changes in HAS presence, n/N: n=number of patients with infarct swelling.

d.f., degree of freedom; HAS, hyperdense artery sign; NA, not applicable.

Of 673 patients with baseline HAS, alteplase reduced persistent HAS (152/350, 43.4%, allocated alteplase vs 172/323, 53.3%, allocated control; unadjusted OR, 0.67 (95% CI 0.50 to 0.91); p=0.01). Patients with higher NIHSS (median, 17 (IQR 13–21) vs median, 14 (IQR 9–19); p<0.001) and large to very large infarcts (152/272, 55.9% vs 172/401, 42.9%; p<0.001) were more likely to have persistent HAS. Patients whose HAS disappeared were less likely to develop symptomatic infarct swelling than those with persistent HAS on follow-up (14/349, 4.0%, disappeared HAS vs 46/324, 14.2%, persistent HAS; unadjusted OR, 0.25 (95% CI 0.14 to 0.47); p<0.001).

Of 2057 patients without baseline HAS, there were fewer newly developed HAS in the alteplase group (90/1047, 8.6%) than the control group (106/1010, 10.5%), although the difference was not statistically significant (unadjusted OR, 0.80 (95% CI 0.60 to 1.09); p=0.16). Patients with newly developed HAS (n=196) on follow-up CT were more likely to develop infarct swelling than the remaining 1861 patients (unadjusted OR, 4.19 (95% CI 2.16 to 8.16); p<0.001).

## Discussion

In this IST-3 cohort of 2961 patients with baseline CT, the presence of HAS, either on baseline CT, or persisting or newly developed on follow-up CT, was associated with a higher risk of developing symptomatic infarct swelling ≤7 days after stroke. Alteplase increased the risk of developing infarct swelling at follow-up whether or not HAS was present at baseline. However, at follow-up, alteplase promoted HAS disappearance in patients with baseline HAS and reduced development of new HAS in those without baseline HAS, and these patients then had less risk of infarct swelling. These findings provide further evidence of benefits of alteplase in not only removing thrombus but also reducing secondary adverse effects of stroke, thereby altering the natural history of ischaemic lesion progression towards a better chance of recovery. Alteplase became a World Health Organisation Essential Medicine in 2018. It is widely used over the world in patients with acute ischaemic stroke and is available for patients who do not meet criteria for thrombectomy, where thrombectomy is unavailable, and as adjunct therapy to thrombectomy. Therefore, these results are relevant to a large proportion of patients with acute ischaemic stroke.

Previous studies reported that HAS at baseline was associated with infarct swelling in patients with stroke treated with alteplase.^27 28^ We have clarified that HAS increases symptomatic infarct swelling in both patients allocated alteplase and those allocated control, and separately, that alteplase increases symptomatic infarct swelling regardless of the presence or absence of baseline HAS. In this large cohort, baseline HAS showed moderate sensitivity and specificity for development of symptomatic infarct swelling, indicating that HAS is just one of several predictors that contributes to swelling. The presence of HAS is associated with more severe stroke and larger infarct size,^29 30^ where the latter two also predict symptomatic infarct swelling.^3 31^ We further show that HAS increases symptomatic infarct swelling independent of NIHSS and infarct size. Our results also suggest that HAS in patients with NIHSS <15 may be a useful warning of increased risk of symptomatic infarct swelling, that is, in patients who may otherwise not be expected to develop this complication, but this requires further testing. In addition, we confirm that older patients are less likely to develop symptomatic infarct swelling independent of HAS, NIHSS, large infarct size and alteplase, possibly attributable to the buffering space for swelling provided by the age-related brain atrophy.

Although alteplase promoted HAS disappearance and improved functional outcome,^17^ we show that it increases symptomatic infarct swelling in general, independent of HAS presence. However, in patients with baseline HAS, who therefore have a high risk of symptomatic infarct swelling, alteplase promoted HAS disappearance and thus reduced symptomatic infarct swelling. Therefore, it appears that the increased risk of symptomatic infarct swelling by alteplase was outweighed by the benefit that resulted from its ability to accelerate racanalisation in this patient group with baseline HAS. Despite this benefit, some patients with baseline HAS allocated to alteplase had the persistent risk of swelling, which may be partly due to unsuccessful reperfusion. Similar findings were reported in the MR CLEAN trial, where successful recanalisation but not the allocation to endovascular treatment was associated with a lower risk of midline shift on follow-up scans.^13^ Although thrombectomy may provide a higher proportion of recanalisation and some patients in IST-3 described in the present analysis would now be candidates for thrombectomy, intravenous alteplase is more accessible than thrombectomy for stroke patients in many areas and should not be withheld from patients presenting with HAS even where thrombectomy is being considered.^32^ Our findings from the IST-3, together with the benefit shown in the MR CLEAN trial, provide evidence that patients at risk of infarct swelling could benefit from reperfusion therapies, either by intravenous thrombolysis or mechanical clot retrieval.

Our study has some limitations. First, we assessed HAS qualitatively on non-contrast CT without HU measurement, since the visual assessment of HAS is clinically relevant and highly specific for angiographic obstruction with good reliability across a wide range of raters,^4–6^ and that HU measurement does not improve sensitivity or specificity.^22 23^ Second, we did not verify the association between HAS and angiographic arterial obstruction for current analysis, because we do not have angiography for all of these patients and notwithstanding, we wish to focus on the imaging sign on non-contrast CT—a technique that is widely accessible and rapidly translatable in clinical practice, and since many patients, in many parts of the world, for many reasons, do not receive routine CT angiography. Therefore, our findings could translate immediately to the generality of stroke worldwide.

## Conclusion

Patients with HAS on non-contrast CT ≤48 hours after stroke onset were more likely to develop symptomatic infarct swelling within 7 days after stroke. Overall, intravenous alteplase increased the risk of symptomatic infarct swelling, but this risk reduced in patients whose baseline HAS had disappeared at follow-up, likely mediated by removal of obstructing thrombus and improved tissue reperfusion, consistent with the clinical benefit of intravenous alteplase.

## Data Availability

The IST-3 data are deposited at https://doi.org/10.7488/ds/1350, available on application to the study investigators.
