# Supplementary material for: Hyperdense artery sign, symptomatic infarct swelling and effect of alteplase in acute ischaemic stroke
Source: Stroke Vasc Neurol. 2020 Nov 27;6(2):238–43. doi: 10.1136/svn-2020-000569 (PMC8258046; doi:10.1136/svn-2020-000569)
Supplement: Supplementary data [file svn-2020-000569supp001.pdf]

SUPPLEMENTAL MATERIAL

Hyperdense artery sign, symptomatic infarct swelling and effect of alteplase in acute ischaemic stroke

Appendix I. IST-3 Collaborative Group

For a complete list of all committees, please see the IST-3 primary publication in The Lancet (The benefits and harms of intravenous thrombolysis with recombinant tissue plasminogen activator within 6 h of acute ischemic stroke (the third international stroke trial [IST-3]): a randomized controlled trial. *Lancet* 2012;379:2352-63).

CO-INVESTIGATORS

Peter Sandercock, DM (University of Edinburgh, Scotland, co-chief Investigator); Richard I Lindley, MD (Sydney Medical School – Westmead Hospital and The George Institute for Global Health, University of Sydney, Australia, co-chief Investigator); and Joanna M Wardlaw, MD (University of Edinburgh, Scotland, co-chief Investigator for neuroradiology).

CONTRIBUTORS

Trial Steering Committee

Colin Baigent (University of Oxford, UK, independent chairman); David Chadwick (University of Liverpool, UK, independent chairman); Pippa Tyrrell (University of Manchester, UK, independent member); Gordon Lowe (University of Glasgow, UK, independent member); Martin Dennis (University of Edinburgh, Scotland); Geoff Cohen (University of Edinburgh, Scotland, statistician); Karen Innes (University of Edinburgh, Scotland, trial coordinator); Heather Goodare (lay representative).

National coordinators and associate national coordinators

Australia: Richard I Lindley, MD (Sydney Medical School – Westmead Hospital and The George Institute for Global Health, University of Sydney, Australia); Graeme J Hankey (Royal Perth Hospital, Perth). Austria: Karl Matz (Landesklinikum Donauregion Tulln, Tulln), Michael Brainin. Belgium: AP. Canada: Gord Gubitz (Dalhousie University and Queen Elizabeth II Health Sciences Centre, Halifax), Stephen J Phillips (Dalhousie University and Queen Elizabeth II Health Sciences Centre, Halifax). Italy: Stefano Ricci (Department of Neurology ASL1, Ospedale, Citta' di Castello). Mexico: Antonio Arauz (Instituto Nacional de Neurologia, Mexico City). Norway: Eivind Berge (Oslo University Hospital, Oslo), Karsten Bruins Slot (Oslo University Hospital, Oslo). Poland: Anna Czlonkowska (Institute of Psychiatry and Neurology, Warsaw, and Medical University of Warsaw, Warsaw), Adam

Wu S, *et al. Stroke Vasc Neurol* 2021; 6:e000569. doi: 10.1136/svn-2020-000569

Kobayashi (Institute of Psychiatry and Neurology, Warsaw, Poland). Portugal: Manuel Correia (Hospital Geral de Santo Antonio, Porto). Switzerland: Phillippe Lyrer (University Hospital Basel, Basel), Stefan Engelter. Sweden: Veronica Murray (Karolinska Institutet, Stockholm), Andreas Terent, Bo Norrving, Per Wester: UK: Graham Venables (Sheffield Teaching Hospitals NHS Foundation Trust, Sheffield, UK).

CT and MRI reading panel

Joanna M Wardlaw, Andrew Farrall (University of Edinburgh, Scotland), Zoe Morris (University of Edinburgh, Scotland), Rüdiger von Kummer (Dresden University Stroke Centre, Germany), Lesley Cala (University of Western Australia, Crawley, Australia), Anders von Heijne (Dandyred Hospital, Stockholm, Sweden), Alessandro Adami (Sacro Cuore-Don Calabria Hospital, Verona, Italy), Andre Peeters (Cliniques Universitaires Saint-Luc, Bruxelles, Belgium), Gillian Potter (Salford Royal NHS Foundation Trust, England), Nick Bradey (Neuroradiology, James Cook University Hospital, South Tees Hospital NHS Trust, Middlesborough, UK).

IST-3 collaborative group and participating hospitals in each country

Figures in parentheses are the number of patients recruited in the country or by the centre.

***UK** (1447)* Royal Hallamshire Hospital (118): G Venables, C Blank, H Bowler, C Doyle, K Endean, K Harkness, E Parker, M Randall. University Hospital of North Staffordshire (97): C Roffe, N Ahmad, A Arora, S Brammer, J Chembala, B Davies, S Ellis, E Epstein, K Finney, C Jackson, C Jadun, R Kinston, H Maguire, I Memon, I Natarajan, M Poulson, R Sanyal, S Sills, A Vreeburg, E Ward. Western General Hospital (95): P Sandercock, R Al-Shahi Salman, R Davenport, M Dennis, P Hand, S Hart, I Kane, S Keir, M MacLeod, L McKinlay, H Milligan, E Sandeman, J Stone, C Sudlow, P Taylor, J Wardlaw, C Warlow, W Whiteley, A Williams. The National Hospital for Neurology & Neurosurgery (84): M Brown, B Athwal, V Bassan, N Bhupathiraju, J Bowler, C Davie, D Doig, R Erande, S Gilbert, L Ginsberg, R Greenwood, S Gregoire, N Harding, N Losseff, R Luder, N Passeron, R Perry, P Rayson, R Simister, S Stone, D Werring. Arrowe Park Hospital (83): J Barrett, H Aitken, S Cherian, R Davis, S Downham, L Godd, V Gott, D Jose, V Little, D Lowe, L Luxford, M McGrory, P Owings, N Price, J Richards, G Sangster, J Sherlock, S Vargese, I Wakefield, P Weir. Southend University Hospital (77): P Guyler, T Attygale, S Chandler, L Coward, S Feasey, C Khuoge, T Loganathan, S Martin, A O'Brien, D Sinha, V Thompson, S Tysoe, R Walsh. Norfolk and Norwich University Hospital (67): K Metcalf, J Cochius, R Fulcher, N Gange, C Green, J Jagger, M Lee, P Myint, J Potter, G Ravenhill, S Shields, N Shinh, T Staunton, E Thomas, W Woodward, P Worth, N Wyatt. Nottingham City Hospital (63): W Sunman, P Bath, P Berman, J Clarke, C Gaynor, F Hammonds, R Harwood, K Mitchell, S Munshi, S Pacey, A Shetty, N Sprigg, H Stear, G Subramanian, A Wills. Guy's & St.Thomas Hospital (60): A Rudd, H Audebert, A Bhalla, J Birns, R Chowdhury, G Cluckie, I Davies, C Gibbs, P Holmes, N Mitchell, F Schiavone, E White, M Yeung. Darlington and Bishop

Auckland Hospitals (56): A Mehrzad, V Baliga, E Brown, L Burnside, B Esisi, J Kent, P Orr, D Stead, E Wayman. University Hospital Aintree (46): R Durairaj, C Cullen, R Kumar, H Martin, D McDowell, A Sharma, V Sutton, R White. University Hospital of Wales (46): T Hughes, K Ali, J Anderson, K Baker, K Bethune, K Bethune, M Booth, M Cossburn, S Halpin, M Hourihan, E Marsh, K Peall, R Powell, H Shetty, M Wardle, M Williams. Derby Royal Hospital (37): K Muhiddin, J Beavan, M Clarke, R Donneley, S Elliott, P Fox, P Gorman, M Harper, M Mangoyana, I Memon, L Mills, L Wright. Addenbrookes Hospital (34): L Warburton, J Baron, P Barry, D Day, T Harold, P Martin, J Mitchell, E O'Brien, J Rycarte, M Turnham. St George's Healthcare NHS Trust (34): G Cloud, L Choy, B Clarke, C Griffin, O Halse, I Jones, F Kennedy, U Khan, R Lewis, A Loosemore, C Lovelock, H Markus, B Moynihan, J O'Reilly, O Paul, A Pereira, M Punter, P Rich, D Rolfe, F Schiavone. Royal Devon & Exeter Hospital (Wonford) (30): M James, J Bell, A Bowring, L Boxall, J Cageao, H Eastwood, S Elyas, F Hall, S Harries, A Hemsley, S Jackson, S Keenan, P Mudd, A Sekhar, D Strain, J Sword, N Wedge. Aberdeen Royal Infirmary (26): M MacLeod, M Bruce, A Joyson, M Kemp, K McMullan, J Reid, O Robb, J Webster, S Wilkinson. Hammersmith Hospitals & Imperial College (24): P Sharma, P Bentley, H Jenkins, A Kar, T Sachs. Northwick Park Hospital (20): D Cohen, R Bathula, J Devine, M Mpelembue. William Harvey Hospital (20): D Hargroves, I Balogun, L Cowie, A Maidment, D Rand, J Rowe, H Rudenko, D Smithard, L Wray. Scarborough Hospital (17): J Paterson, J Brown, J Hampton, S Jamieson, R Rose, A Volans. Countess of Chester Hospital NHS Foundation Trust (17): K Chatterjee, G Abbott, R Brookes, C Castle, C Kelly, S Leason, A Nallasivan, A Sen. Watford General Hospital (17): D Collas, M Cottle, N Damani, P Jacob, D Oza, D Werring. University Hospitals Coventry & Warwickshire NHS Trust (15): A Kenton, N Adab, L Aldridge, H Allroggen, Y Brown, R Cross, L Galvin, K Ghosh, A Grubneac, A Lindahl, H Mehta, M Pritchard, C Randall, P Ray, A Shehu, S Thelwell. Royal Bournemouth & Christchurch NHS Trust (12): D Jenkinson, J Bell, T Black, O David, J Kwan, A Orpen, C Ovington, D Tiwari, Z ud Din Babar. Leeds General Infirmary (12): A Hassan, A Bailey, J Bamford, C Bedford, R Bellfield, J Cooper, L Dunsmure, J Greig, M Keeling, L Mandizvidza, J Rankine, E Roberts, P Wanklyn, T Webb, S Williamson. York Health Services NHS Trust (12): J Coyle, S Crane, C Croser, P Duffey, R Evans, E Iveson, M Keeling, G Kitching, M Porte, C Rhymes. Queen Elizabeth Hospital (Gateshead) (12): D Barer, M Armstrong, M Bokhari, T Cassidy, B McClelland. Queen Elizabeth The Queen Mother Hospital (10): G Gunathilagan, P DOLKE, S Jain, S Jones, A Maidment, L Rosser, G Thomas, C White. Worcestershire Royal Hospital (10): P Sanmuganathan, C Scholtz, E Stratford. Blackpool Victoria Hospital (10): M O'Donnell, H Goddard, G Hoadley, J Howard, S Leach, J McIlmoyle, A Stewart, A Strain. Basildon & Thurrock University Hospitals NHS FT (9): F Huwez, P Croot, N Gadi, N Mguni, U Umasankar. Royal Infirmary of Edinburgh (8): G Mead, B Chapman, A Coull, S Hart, A Kinnear, B Morrow, F Morrow. St Mary's Hospital (8): D Ames, J Ball, S Bannerjee, J Chataway. Yeovil District Hospital (8): K Rashed, C Buckley, D Donaldson, D Hayward, C Lawson. Luton and Dunstable Hospital (8): L Sekaran, K Bharaj, F Justin, G Jutlla, D Phiri, S Sethuraman, M Tate. Solihull

Hospital, Heart of England NHS Trust (8): D Sandler, P Carr, G Jones, J Lyons, K Warren. King's College Hospital (7): L Kalra, A Davis, J Jarosz, D Manawadu, L Sztriha. Doncaster Royal Infirmary (7): D Chadha, A Holford, P Willcoxson. Royal United Hospital Bath (7): L Shaw, D Button, A Cunningham, L Dow, J Dutson, T Hall, C Hardy, N Jakeman, P Kaye, B Madigan, K O'Brien, D Pressdee, M Price, L Robinson, C Taylor, D Williamson. Birmingham Heartlands Hospital (6): D Sandler, P Carr, J Lyons, J McCormack, C Stretton. University Hospital North Durham (6): P Earnshaw, E Brown, S Bruce, C Church, S Desai, B Esisi, M Myint, N Watt. Wansbeck General Hospital (6): C Price, S Elliott, H Graham, R Lakey, K Mitchelson. Bristol Royal Infirmary (6): P Murphy, L Ball, S Caine, J Dovey, J Hughes, A Steele. Stepping Hill Hospital (6): K Dizayee, A Brown, T Chattopadhyay, J Cheetham, H Cochrane, A Datta, M Datta-chaudhuri, C Fox, D Kilroy, S Krishnamoorthy, F Levy, S Metha, P Ngoma, B Venkatesh. Princess Royal Hospital Brighton & Sussex University Hospitals Trust (5): K Ali, R Gautam, N Henderson, M Jones, S Murphy, G Spurling. Belfast City Hospital (5): I Wiggam, C Boyd, K Fullerton, P Gray, M Kinnaird, S MacNair, C Morgan, M Reid, S Tauro. Royal Liverpool University Hospital (5): S Loharuka, D Balmforth, P Cox, G Fletcher, A Ledger, A Manoj, M Wilkinson. City Hospital, Sandwell & West Birmingham Hospital (5): D Nicholl, S Clegg, S Hurdowar, S Kausar, K Law, A Singal, S Sturman. Royal London Hospital (4): P Gompertz, J Evanson, A Farrell, A Petrou, K Saastamoinen, T Sachs, A Salek-Haddadi, R Yadava. Sunderland Royal Hospital (4): J O'Connell, H Brew, S Butler, S Crawford, C Gray, D Gulliver, N Majmudar, R O'Brien. Morriston Hospital (4): M Wani, L Dacey, L Davies, R Evans, D Harris, T Jones, S Storton. Royal Preston Hospital (4): S Punekar, A Ashton, S Duberley, H Emsley, C Gilmour, B Gregary, L Hough, S Philip, S Wuppalapati. The Royal Wolverhampton Hospitals NHS Trust (4): K Fotherby, P Bourke, D D'Costa, K Kauldhar, D Leung, R Lodwick, S McBride, D Morgan, M Qaiyum, G Sahota, M Srinvasan. Royal West Sussex NHS Trust, St Richard's Hospital (4): I Kane, N Chuter, L Garrad, M Hookway, S Ivatts, G Kennedy. Queen's Hospital Romford (4): K Darawil, L Al Dhahirl, S Andole, M Baig, P Dugh, K Dunne, H Kariuki, M Khan, S Rathnayaka. Ulster Hospital (3): M Power, K Dynan, J Finnerty, A Heaney, C Leonard, K McKnight, J Turkington, B Wroath. Great Western Hospital (3): B Dewan, S Cotton, M Gardiner, T Saunders, B Vincent. The Queen Elizabeth Hospital Birmingham (3): D Sims, P Guest, E Jones, J McCormack, D Nicholl, J Savanhu, R Tongue, M Willmot. Leicester General Hospital (3): D Eveson, S Dawson, M Dickens, M Fotherby, R Hunt, S Khan, T Kumar, R Marsh, A Mistri, T Robinson, J Thompson. Darent Valley Hospital, Dartford & Gravesham NHS Trust (3): P Aghoram, T Daniel, M Gatehouse, S Hussein, A Jackson, T Shanganya, E Strachan, G Tan. Nevill Hall Hospital, Aneurin Bevan Local Health (3): B Richard, S Elaine, S Hanson, S Mosely, H Reed, M Williams. Colchester Hospital University Foundation Trust (3): R Saksena, S Cook, D Demuran, M Keating, R Needle, V Paramsothy, A Sebastian, R Sivakumar, A Wright. Salford Royal Hospital Foundation NHS Trust (2): R Grue, E Barberan, C Dickson, C Douglas, J Jellicoe, T Marsden, J Priestley, E Quick, C Sherrington, A Singh, C Smith, J Stevens, P Tyrell, J Wainwright. Leicester

Wu S, *et al. Stroke Vasc Neurol* 2021; 6:e000569. doi: 10.1136/svn-2020-000569

Royal Infirmary (2): M Ardron, J Birchall. Queen Elizabeth Hospital (Kings Lynn) (2): R Shekhar, C Barsted, S Coleman, S Fletcher, J Graham. John Radcliffe Hospital (2): A Buchan, J Hinkle, J Kennedy, A Manoj, M Westwood. Derriford Hospital (2): A Mohd Nor, S Allder, B Hyams, A Pace. West Cumberland Hospital (1): E Orugun, C Brewer, L Huntley, R Jolly, C Summers. Sandwell General Hospital (1): K Sharobeem, J Khaira, J Leahy, E Linehan, G Moore, J Rizkalla, J Wilkinson. Torbay Hospital (1): D Kelly, C Hilaire. Warrington & Halton Hospitals NHS Foundation Trust (1): O Otaiku, L Connell, G Delaney-Sagar, G James, L Lomax, D Matthew, J Simpson, H Whittle. Medway Maritime Hospital (1): S Sanmuganathan, S Burrows, A Mahmood. Southampton General Hospital (1): G Durward, S Barker, J Cantle, P Crawford, S Evans, V Pressly, N Weir. Victoria Hospital (1): V Cvoro, K McCormick. ***Poland*** *(347)* 2nd Department of Neurology, Institute of Psychiatry & Neurology (190): A Czlonkowska, J Bembenek, M Bilik, G Chabik, W Czepiel, J Dzierka, M Gluszkiewicz, K Grabska, B Janus-Laszuk, J Jedrzejewska, A Kobayashi, T Litwin, A Oskedra, A Piorkowska, M Skowronska, A Sliwinska, U Stepien. SPZZOZ w Sandomierzu (43): P Sobolewski, A Gajewska, M Grzesik, R Hatalska-Zerebiec, I Labudzka, B Loch, A Medrykowska, M Sledzinska, A Sobota, W Szczuchniak, G Wolak, I Zdyb. Medical University of Gdansk (35): W Nyka, D Gasecki, K Chwojnicki, A Gojska, B Karaszewski, G Kozera, M Kwarciany, M Nowak, M Swierkocka-Miastkowska , S Szczyrba, M Wisniewska, E Wnorowska. 1st Department of Neurologyt, Institute of Psychiatry & Neurology (25): P Richter, A Bochynska, M Chahwan, A Graban, R Rola. Military Medical Institute (24): A Stepien, B Brodacki, M Grotowska, J Kotowicz, J Staszewski, J Swistak, S Zaloga. Szpital Powiatowy (14): J Stoiñski, K Czajkowaka-Fornal, P Czubak, A Kaczor, J Kraska, E Nowakowska-Sledz, J Ozdoba-Rot, E Zawadzka. Szpital Specjalistyczny w Konskich (8): M Fudala, D Adamczyk, W Brola, I Guldzinska, K Kaluzny, M Kucharska-Lipowska, M Mosiolek, M Polewczyk, M Ziomek. Central University Hospital (7): G Opala, M Arkuszewski, M Kudlacik, P Malgorzata, M Swiat. SPSK im. Prof. W. Orlowskiego CMKP (1): U Fiszer, M Lenska-Mieciek. ***Italy*** *(326)* Ospedale Di Citta' di Castello (62): S Cenciarelli, A Barilaro, R Condurso, F Coppola, S Dioguardi, E Gallinella, A Mattioni, C Menichetti, S Ricci. Nuovo Ospedale Civile "S.Agostino-Estense" (40): F Casoni, M Bacchelli, M Cavazzuti, M Malagoli, A Zini. Ospedale Beato Giacomo Villa - Citta' della Pieve (36): G Benemio, M Celani, R Allegrucci, V Bondo, S Cupella, L Guerra, S Guerrieri, C Ottaviani, E Righetti, C Rossi, N Sacchi, M Scucchi, V Stefanini. Ospedale Di Branca (36): T Mazzoli, A Bigaroni, L Greco, R Paris, P Parise, S Ricci. A.O. Niguarda Ca'Granda (25): A Ciccone, L Basso, R Causarano, P Doneda, E Ferrante, A Gatti, A Guccione, A Gullo, F Imbesi, S Jann, R Marazzi, E Moro, C Motto, D Parodi , A Protti, M Riva, A Rosiello, I Santilli, R Sterzi, P Tiraboschi, G Venturelli. R.Guzzardi Hospital - Vittoria (RG) (19): F Iemolo, R Campagna, G Campagnolo, A Carnemolla, N D'Apico, G D'Asta, S Giannarita, A Giordano, E Sanzar. Ospedale Regionale di Aosta (17): E Bottacchi, S Cordera, G Corso, M Di Giovanni, G Giardini, C Lia, T Meloni, M Pesenti Campagnoni, P Tosi. Ospedale Sacro Cuore - Negrar Verona (12): A Adami, G Rossato, T Zuppini. Ospedale Maggiore - Bologna (12): G Procaccianti, T

Sacquegna. Universita degli Studi di Genova (11): C Gandolfo, M Balestrino, C Bruno, L Castellan, M Del Sette, A Ferrari, C Finocchi, N Reale, D Rizzi. Ospedale Civlle S.Andrea (9): M Del Sette, L Benedetti, C Capellini, E Carabelli, E Cibei, M Godani, G Guariglia, E Landini, A Mannironi, B Nucciarone, S Parodi, S Tonelli, E Traverso, D Zito. Ospedale S. Giovanni Battista - Foligno (8): P Brustenghi, F Corea, O Flamini, S Lolli, G Pelliccia, R Ricci, S Stefanucci, M Zampolini. Ospedale a Vibo Valentia (8): D Consoli, F Galati, P Postorino. Azienda Ospedaliero-Universitaria "Ospedali Riuniti" di Foggia (8): G Rinaldi, E Carapelle, G Grilli, M Guido, L Specchio. Ospedale Valduce di Como (8): N Checcarelli, G Borin, L Chiveri, R Clerici, E Corengia, L Gandola, P Garavaglia, M Guidotti, A Martegani, M Mauri, F Muscia, F Raudino. Ospedale di Cattinara - Trieste (4): F Chiodo Grandi, A Bratina, N Carraro, M Gaio, A Granato, N Koscica, M Naccarato, V Sarra, P Schincariol, C Vilotti, Z Zugna. Clinica Dr Pederzoli Spa (4): D Idone, C Bonato, E De Angelis, A Forgione, M Gambera, F Recchia, S Tamburin, P Tinazzi Martini, G Zanette. Ospedale Civile San Matteo Degli Infermi - Spoleto (2): S Grasselli. Ospedale Silvestrini - Perugia (2): G Agnelli, A Andrea, A Billecia, V Caso, V Casso, R Fabiola, P Fanelli, M Paciaroni, B Sergio, M Vemti. Mater Salutis Hospital, Legnago VR (2): M Silvestri, L Altarini, A Bonfante, M Bonornetti, B Costa, N D'Attoma, N Deluca, F Frattini, R Niego, D Rafaele, V Ravenna, M Turazzini. Ospedale Guglielmo da Saliceto - Piacenza (1): S Cammarata. ***Sweden*** *(297)* Uppsala University Hospital (100): E Lundström, L Jonsson , U Söderström, A Terént. Danderyd Hospital (46): V Murray, A Alvelius, M Arbin von, I Dalenbring, Å Doverhall, Å Franzén-Dahlin, N Greilert, M Hallberg, A Heijne von, E Isaksson, H Kumpulainen, A Laska, A Lundström, C Martin, J Muhrbeck, E Näslund, N Ringart, E Rooth, R Undén, P Waldenström. Hassleholm Hospital (29): M Esbjornsson, M Petranek. Karnsjukhuset (25): B Cederin, E Bertholds, A Elgåsen, T Johansson, B Witteborn. Koping Hospital (20): M Kwiatkowska, E Gustafsson, T Noren, J Saaf. Mora Hospital (17): J Teichert, M Bertilsson, S Nilsson, S Oestberg. LidkopingHospital (11): L Welin, K Fredricson, L Pehn. Falu Hospital (11): J Hambraeus, I Lonn. Capio S: tGoran Hospital (9): B Hojeberg, A Adolfsson, M Anzen. Vastervik Hospital (5): T Wallen, R Schloenzig, P Söderström, A Wennerberg. University Hospital MAS (5): F Buchwald, K Abul-Kasim, A Berkeskold, J Petersson, E Poromaa. University Hospital of Northern Sweden (4): P Wester, R Backlund, A Sjöström. Helsingborgs lasarett (4): B Hedström , E Campbell, K Johnsson, B Karlsson, N Lekokotla, C Lundahl, A Risedal, P Sandgren, A Svensson. Visby Hospital (4): S Bysell, E Smedberg, A Vestberg Bysell. Sundsvall Hospital (3): V Sjögren, B Högvall. University Hospital Lund (2): G Andsberg, T Cronberg, A Lindgren. Vasteras Hospital (1): H Wannberg, F Ax, L Nyren. Karlstad Central Hospital (1): J Sanner, H Andersson, F Andler, S Holmgård, R Johansson, I Magnussan, K Nilsson, J Rådberg. ***Norway*** *(204)* Trondheim University Hospital (69): B Indredavik, H Ellekjær, A Østvik, G Rohweder, D Steckhan, J Storvold. Oslo University Hospital Sykehus (66): E Berge, Y Rønning, R Aakvik, K Bruins Slot, G Knutsen, M Moxness, R Pettersen, T Wyller. University Hospital of North Norway (23): C Wahl, O Iversen, S Johnsen, B Norderhus, L Steffensen, E

Stensland. Kongsvinger Hospital (13): T Asak, J Aaseth, T Rotnes, J Sparby, S Wetterhus. Levanger Hospital (9): H Hallan, A Aardal, T Graven, H Hansbakk Skjetne, B Klykken, K Lindqvist, A Tommy. University Hospital of North Norway (8): T Engstad, M Antonsen, R Bajic, W Fønnebø, S Hykkerud, I Lyngmo, A Nyrnes, S Rogne, S Sparr. Harstad Hospital (7): O Kildahl-Andersen, K Pedersen, H Ulrichsen. Ålesund Hospital (4): O Skogen, I Alnes, R Hukari, Y Seljeseth, P Vadset. Asker and Bærum Hospital (2): G Knutsen, B Fure, H Ihle-Hansen, N Johnsen, L Kornberg. Namsos Hospital (2): S Schuler, M Heibert. Volda Hospital (1): M Lillebø, O Aasen, I Eskeland, T Hamre, S Hareide, H Helset, K Kolnes, B Lødemel, H Ose Velle, S Reite, E Velle. ***Australia** (179)* Nambour General Hospital (51): R Grimley, E Ahern, C Cocks, M Courtney, R Devin, J Endacott , C Fawcett , V Harrington, C Johnston, M Koltermann, S Murray, K Ng, G Styles, A Tampiyappa. John Hunter Hospital (29): C Levi, K Chung, L Dark, M Evans, Y Gawarikar, E Kerr, A Loiselle, F Miteff, A Moore, W O'Brien, M Parsons, D Quain, A Royan, M Russell, N Spratt. Gosford Hospital (24): J Sturm, D Crimmins, D Griffiths, P Kavelieros, J Kinsella, A Malhotra, B O'Brien, A Schutz, M Webb, S Whyte, V Zenteno. Westmead Hospital (16): R Lindley, A Bleasel, N Cordato, A Duggins, V Fung, L Gomes, N Ingham, J Ip, P Landau, J Morris, S Vucic. Royal Perth Hospital (15): G Hankey, A Claxton, N Lillywhite. The Canberra Hospital (12): C Lueck, C Andrews, G Danta, C Das, I Harvey, A Hughes, C McColl, A Oon, R Tuck. Royal Brisbane and Women's Hospital (10): S Read, M Badve , M Broad, G Cadigan, H Cavanagh, J Chalk, D Copsinis, K Etherington, R Henderson, R Hull, J O'Sullivan, J Pandian, L Ross-Lee, M Roxas, N Sheikh, G Skinner, A Wong. Austin Health - Repatriation Campus (8): H Dewey, A Brodtmann, G Donnan, A Hughes, M Karonen, H Ma, T Mulcahy, S Petrolo, L Walker, D Young, J Zavala. Nepean Hospital (7): M Thieben, C Harris, M Krause, S Lane, H Park, M Shaffi, J Wood. Box Hill Hospital (7): C Bladin, A Buckland, K Coughlan, B Coulton, A Gilligan, P Lee, S Mullen, Z Ross, P Sien Loh, C Szoeke. ***Portugal** (82)* UAVC. Centro Hospitalar de Trás-os-Montes e Alto Douro (42): M Silva, F Afonso, J Gabriel, P Guimarães, A Velon. Hospital Pero da Covilhã (19): M Castelo- Branco, F Alvarez, V Branco, C Coxo, P Goulao, D Leal, S Morgado, R Oliveira, F Paiva, A Rodrigues, M Simoes. Hospital de Santo António (12): G Lopes, T Almeida, M Cardoso, J Chaves, C Correia, M Correia, J Damásio, R Felgueiras, J Pereira, A Tuna. Hospital S.Marcos (9): C Ferreira, E Lourenco, A Machado, R Mare, J Rocha. ***Belgium** (73)* Cliniques Universitaires St Luc (73): A Peeters. ***Austria** (46)* Landesklinikum Donauregion Tulln (34): K Matz, M Brainin, G Funk, V Reiner-Deitemyer. Krankenhaus Der Barmherzigen Bruder Wien (9): J Ferrari, A Flamm-Horak, G Gruber, R Rattinger. Krankenhaus Göttlicher Heiland (3): W Muellbacher, D Doppelbauer, R Kalchmayr, W Schima, T Wieser, M Zart. ***Switzerland** (23)* Universitatsspital Basel (22): P Lyrer, L Bonati, S Engelter, F Fluri, S Muller, E Radue, A Tiemessen, L Walz, F Weisskopf, S Wetzel. Universitätsspital Zürich (1): A Luft, D Fetz, B Hertler, A Pangalu. ***Canada** (8)* QEII Health Sciences Centre (8): G Gubitz, P Boulton, J Jarrett, J Moeller, S Phillips. ***Mexico** (3)* Instituto Nacional de Neurologia y Neurojrugia MVS (3): A Arauz, L Bermudez, J Calleja, R Garcia.

Appendix II. Funding sources for IST-3

The startup phase of IST-3 was supported by a grant from the Stroke Association, UK (TSA 04/99; Richard Lindley, Peter Sandercock, Joanna Wardlaw). The expansion phase was funded by the Health Foundation, UK (2268/1282; Peter Sandercock, Joanna Wardlaw). The main phase of the trial is funded by UK Medical Research Council (MRC) (grant numbers G0400069 and EME 09-800-15) and managed by NIHR on behalf of the MRC-NIHR partnership (Peter Sandercock, Joanna Wardlaw); the Research Council of Norway (Eivind Berge); Arbetsmarknadens Partners Forsakringsbolag (AFA) Insurances Sweden (Veronica Murray (deceased)); the Swedish Heart Lung Fund (Veronica Murray (deceased)); The Foundation of Marianne and Marcus Wallenberg, Stockholm County Council (Veronica Murray (deceased)); Karolinska Institute Joint ALF project grants Sweden (Veronica Murray (deceased)); the Polish Ministry of Science and Education (grant number 2PO5B10928; Anna Czlonkowska); the Australian Heart Foundation (Richard Lindley); Australian National Health and Medical Research Council (NHMRC) (Richard Lindley); the Swiss National Research Foundation (Phillippe Lyrer); the Swiss Heart Foundation (Phillippe Lyrer); the Foundation for Health and Cardio-/Neurovascular Research, Basel, Switzerland (Phillippe Lyrer); the Assessorato alla Sanita, Regione dell'Umbria, Italy (Stefano Ricci); and, Danube University, Krems, Austria (Karl Matz). Boehringer-Ingelheim GmbH donated drug and placebo for the 300 patients in the double-blind phase, but thereafter had no role in the trial. The UK Stroke Research Network (SRN study ID 2135) adopted the trial on 1/5/2006, supported the initiation of new UK sites, and in some centers, and, after that date, data collection was undertaken by staff funded by the network or working for associated NHS organizations. IST-3 acknowledges the support of the NIHR Stroke Research Network, NHS Research Scotland (NRS), through the Scottish Stroke Research Network, and the National Institute for Social Care and Health Research Clinical Research Centre (NISCHR CRC). The central imaging work was undertaken at the Brain Imaging Research Centre (www.sbric.ed.ac.uk), a member of the Scottish Imaging Network: A Platform for Scientific Excellence (SINAPSE) collaboration (www.sinapse.ac.uk; Joanna Wardlaw), at the Division of Clinical Neurosciences, University of Edinburgh. SINAPSE is funded by the Scottish Funding Council (SFC; Joanna Wardlaw) and the Chief Scientist Office of the Scottish Executive (CSO; Joanna Wardlaw). The scan reading development was funded by Chest, Heart and Stroke Scotland (R100/7; Joanna Wardlaw) with additional support from DesAcc, and University of Edinburgh.
